# The antinociceptive effect of systemic gabapentin is related to the type of sensitization-induced hyperalgesia

**DOI:** 10.1186/1742-2094-4-15

**Published:** 2007-06-05

**Authors:** M Mar Curros-Criado, Juan F Herrero

**Affiliations:** 1 Departamento de Fisiología, Campus Universitario, Universidad de Alcalá, Alcalá de Henares, 28871 Madrid, Spain

## Abstract

**Background:**

Gabapentin is a structural analogue of gamma-aminobutyric acid with strong anticonvulsant and analgesic activities. Important discrepancies are observed on the effectiveness and potency of gabapentin in acute nociception and sensitization due to inflammation and neuropathy. There is also some controversy in the literature on whether gabapentin is only active in central areas of the nervous system or is also effective in the periphery. This is probably due to the use of different experimental models, routes of administration and types of sensitization. The aim of the present study was to investigate the influence of the spinal cord sensitization on the antinociceptive activity of gabapentin in the absence and in the presence of monoarthritis and neuropathy, using the same experimental protocol of stimulation and the same technique of evaluation of antinociception.

**Methods:**

We studied the antinociceptive effects of iv. gabapentin in spinal cord neuronal responses from adult male Wistar rats using the recording of single motor units technique. Gabapentin was studied in the absence and in the presence of sensitization due to arthritis and neuropathy, combining noxious mechanical and repetitive electrical stimulation (wind-up).

**Results:**

The experiments showed that gabapentin was effective in arthritic (max. effect of 41 ± 15% of control and ID50 of 1,145 ± 14 micromol/kg; 200 mg/kg) and neuropathic rats (max. effect of 20 ± 8% of control and ID50 of 414 ± 27 micromol/kg; 73 mg/kg) but not in normal rats. The phenomenon of wind-up was dose-dependently reduced by gabapentin in neuropathy but not in normal and arthritic rats.

**Conclusion:**

We conclude that systemic gabapentin is a potent and effective antinociceptive agent in sensitization caused by arthritis and neuropathy but not in the absence of sensitization. The potency of the antinociception was directly related to the intensity of sensitization in the present experimental conditions. The effect is mainly located in central areas in neuropathy since wind-up was significantly reduced, however, an action on inflammation-induced sensitized nociceptors is also likely.

## Background

Gabapentin is a structural analogue of gamma-aminobutyric acid with a strong anticonvulsant activity. It is also an effective agent in the treatment of neuropathic pain [1,2] with a mechanism of action, that was initially thought to involve the modulation of GABA-ergic transmission, but that it currently seems more related to the blockade of voltage-gated calcium channels [3-6].

Although gabapentin is an effective analgesic in different types of neuropathies [7,8], important differences are observed when comparing the effectiveness and potency of the drug in studies in humans [9,10] and rodents [11]. Gabapentin also seems to be an effective analgesic drug in some models of inflammatory pain [12,13] but not in all tests [14,15] nor in all studies [11,16-18]. The differences in the results appear to be very dependent on the model of hyperalgesia utilized, the way the drug is administered and the tests used [19]. Opposite results have also been reported when studying the antinociceptive activity of gabapentin in the absence of sensitization, ranging from a facilitation of nociceptive neuronal activity [12] to a virtual full inhibition of responses [20].

There is also some controversy in the literature on whether gabapentin is only active in central areas of the nervous system or whether it is also effective in the periphery [4,14,20-23]. The discrepancies observed might be due to the different techniques used to assess the effect of gabapentin, as well as the different experimental protocols, types of stimulation and models of sensitization.

The aim of the present study was to investigate the influence of the spinal cord sensitization on the antinociceptive activity of gabapentin in the absence and in the presence of monoarthritis or neuropathy, using the same experimental protocol of stimulation and the same technique of evaluation of antinociception. Spinal cord nociceptive activity was elicited by noxious mechanical stimulation and by high intensity repetitive electrical stimulation that triggers the centrally mediated phenomenon of wind-up. Additionally, we examined whether the systemic administration of gabapentin modulates the wind-up phenomenon in the three experimental conditions, in order to distinguish a peripheral from a central action [24].

## Methods

### Animals and induction of sensitization

The antinociceptive activity of gabapentin (Medichem) was studied on 23 adult male Wistar rats (225–380 g) divided into three experimental groups: i) normal animals (n = 6), ii) animals with monoarthritis (n = 8) and iii) animals with mononeuropathy (n = 9). Monoarthritis was induced 16 h before the experiment under halothane anesthesia (5% in oxygen for induction and 2% for maintenance) with an injection of 50 μl carrageenan λ (Sigma, 10 mg/ml, in distilled water) in the right knee cavity. The degree of articular inflammation was assessed by comparing the knee perimeter before the induction of inflammation and immediately prior to the experiment. Mononeuropathy was induced under the same anesthetic regime, seven days before the experiment, using the partial sciatic nerve ligation technique [25]. The development of hyperalgesia in animals with neuropathy was assessed by behavioral experiments, studying withdrawal reflex responses evoked by mechanical and thermal stimulation. Mechanical stimulation was applied by means of Von Frey filaments (60, 80, 100, 200, 300 and 500 mN) following the technique described in detail previously [26,27]. The rats were placed on a raised wire mesh grid under plastic chambers. Each filament was applied ten times for approximately 1 s to the plantar surface of each hind paw in an ascending series and the total number of positive responses was counted. A response was considered positive when a withdrawal of the paw due to the application of the stimulus was observed. Thermal hyperalgesia was assessed by measuring paw withdrawal latencies to 55°C radiant heat generated by an algesimeter (Ugo Basile plantar test; [28]). Two consecutive thermal stimuli were applied to each of the paws with an interval of 2–3 min between tests. In order to avoid tissue damage, a maximum cutoff time was set to 17 s. Tests were made previous to the nerve ligation and at 1, 4 and 7 days after the induction of neuropathy. In all cases, the induction of arthritis and neuropathy produced a significant increment of nociceptive responses that was considered as hyperalgesia due to sensitization.

### Recording of single motor units

The recording of spinal cord neuronal nociceptive responses following the single motor unit (SMU) technique has been described in detail several times [29-33]. Briefly, the preparatory surgery was performed under halothane anesthesia (5% in oxygen for induction and 2% for maintenance) and only consisted of the cannulation of the trachea, two superficial branches of the jugular veins and one carotid artery. One of the veins was used for the continuous administration of α-chloralose, whereas the drugs studied in the experiments were administered through the other branch. The cannulation of the carotid artery was utilized for the monitorization of mean arterial pressure. Halothane was discontinued after surgery and the anesthesia was maintained with α-chloralose (50 mg/kg for induction and 25 mg/kg/h, by perfusion pump, for maintenance in a rate of 1 ml/h to assure a correct animal hydration). Core temperature was maintained at 37 ± 0.5°C by means of feedback controlled blanket. Blood pressure was monitored continuously during the experiments and rats with a systolic pressure below 100 mmHg before the administration of any drug were rejected for the experiment. In all cases the preparation was left to rest for at least one hour after the surgery before any drug was tested.

**Figure 1:**
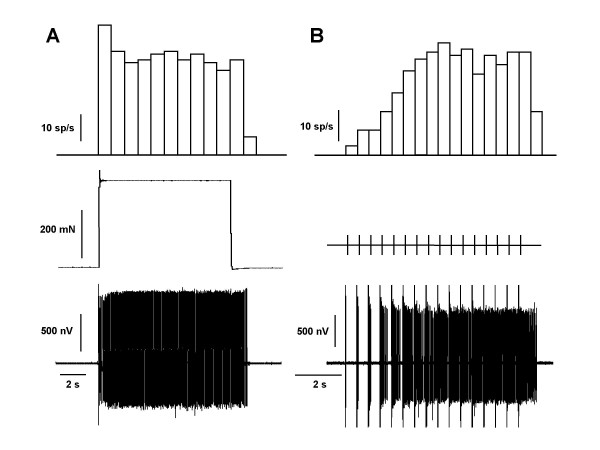
**Original single motor unit spikes**. The figure shows actual spikes (lower panel) recorded during 10 s of noxious mechanical stimulation (A) and a train of 16 electrical stimuli (B; wind-up). Top panel shows the number of spikes/s (sp/s) recorded for each stimulus as bar histograms (mechanical stimulation in mN and electrical pulses as TTL pulses).

Nociceptive activity was elicited in 3 min cycles consisting of 10 s noxious mechanical stimulation and one train of sixteen percutaneous electrical stimuli (2 ms pulse width, 1 Hz and twice the threshold intensity for the recruitment of C-fibers) applied to the most sensitive area of the cutaneous receptive field of the unit. Figures 1 and 3 show examples of the protocol followed in all the experiments. Mechanical stimulation was performed by a computer-controlled pincher device (Estimec, Cibertec, Spain) using a force of 200 mN over the threshold and applied on an area of 14 mm^2^. The threshold force was considered as the minimum force required to trigger a sustained nociceptive reflex over the period of 10 s of stimulation (see Figure 1A for an original recording of spikes recorded with this stimulation). Electrical stimulation was used to study the phenomenon of wind-up (see [24] for review). Data from electrical stimulation were analyzed by counting C-fiber mediated inputs (see Figure 1B for an original recording of the wind-up response). At the end of the experiments the animals were killed with an overdose of sodium pentobarbital (Dolethal, Vetoquinol S.A.). All experiments in this study were undertaken in accordance with Spanish and European Union legislation regarding the uses of animals for experimental protocols and all efforts were made to reduce the number of animals used.

**Figure 2:**
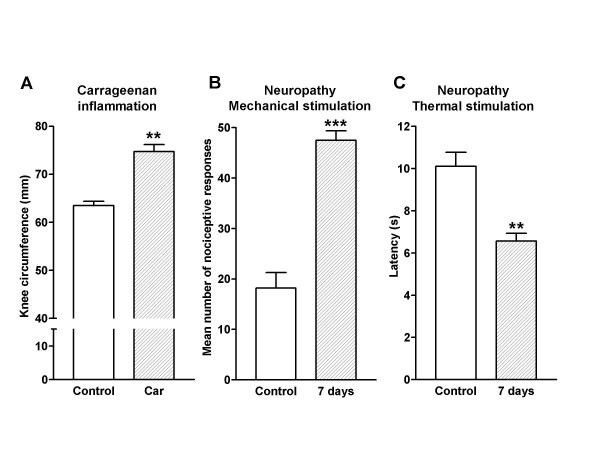
**Induction of sensitization**. (A) The administration of 50 μl of carrageenan in the knee joint induced an evident inflammation. The circumference of the knee increased significantly 16 h after the injection of carrageenan. Neuropathy was induced seven days before the experiment following the partial sciatic nerve ligation technique. (B) Mechanical hyperalgesia was studied by applying a series of von Frey filaments previous to and at days 1, 4 (not shown) and 7 after the nerve ligation. (C) Thermal hyperalgesia was assessed by measuring paw withdrawal latencies to 55°C radiant heat using a similar timing. An intense mechanical and thermal hyperalgesia was observed in all tests made after the induction of neuropathy (**P < 0.01, ***P < 0.001, comparison vs. control response with the two tail unpaired t-test).

**Figure 3:**
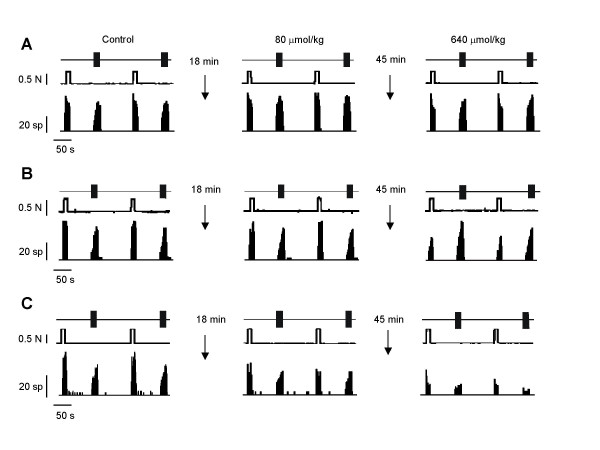
**Original recordings**. Original recordings of three different single motor units previous to and after the administration of iv. cumulative doses of gabapentin in normal (A), arthritic (B) and neuropathic animals (C). The units were activated in three minute cycles by 10 s of noxious mechanical stimulation and 16 electrical pulses (2 ms pulse width, 1 Hz and twice the threshold intensity for the recruitment of C-fibers). Gabapentin was administered iv. in log2 cumulative doses every three cycles of stimulation (9 minutes) from 40 to 1,280 μmol/kg (7 to 224 mg/kg). The administration of gabapentin dose-dependently reduced the nociceptive responses in arthritic and neuropathic animals but not in normal rats.

Gabapentin (Medichem) was dissolved in distilled water 0.5 μmol/μl and diluted in saline. The drug was prepared everyday, immediately before it was administered, and was injected in cumulative log2 regime in a total and constant volume of 0.3 ml. Preliminary experiments showed that peak effect of gabapentin was observed within the first 7 min after intravenous administration. According to this, the doses studied were administered every 3 cycles of stimulation (9 min). The effect of the highest cumulative dose was studied for a minimum of 30 min and no further depression of the responses was observed during this time. The initial dose used was 40 μmol/kg (7 mg/kg) and the highest dose was 1,280 μmol/kg (224 mg/kg; Figure 3 shows the protocol of stimulation, some control responses and the effect of gabapentin observed in the three experimental conditions). Data are presented as percentage of control, control being the average of the responses in the three cycles (number of spikes recorded for each of the stimulus and counted and analyzed separately) previous to the administration of the drug (mean ± s.e.m.). Wind-up responses are presented as actual mean number of spikes or as percentage of control in order to facilitate comparisons. In addition, wind-up-index (total number of spikes/spikes recorded in the first pulse × number of pulses; see [24] for more details and references within) has been considered for the control comparison. The effects of the drug in single motor unit experiments, the comparisons of the effects between experimental conditions and the comparison of regression curves were assessed with the one-way analysis of variance (ANOVA) with post-hoc Tukey's multiple comparison test, whereas the comparison of ID50s, level of inflammation and results in behavioral experiments were made with the two tail unpaired t-test using commercial software (GraphPad Prism and GraphPad InStat).

## Results

The administration of carrageenan in the knee cavity (Figure 2) induced a significant increase of the knee circumference (increment of 18 ± 2%; from 63 ± 0.8 to 74 ± 1.4 mm; P < 0.01). In animals with mononeuropathy the number of nociceptive responses to mechanical stimulation increased from a mean of 18 ± 2 to 47 ± 2 responses per animal (P < 0.001; Figure 2). In addition, the latency to noxious thermal stimulation decreased from 10 ± 0.6 to 6.5 ± 0.4 s (P < 0.01; Figure 2).

In electrophysiological experiments, the mean forces used for mechanical stimulation in the three experimental groups were 0.8 ± 0.1 N in normal animals (mean threshold of 0.65 ± 0.2 N), 0.8 ± 0.1 N in arthritic animals (mean threshold of 0.7 ± 0.1 N), and 0.9 ± 0.1 N in animals with neuropathy (mean threshold of 0.76 ± 0.1 N). The mean intensities of electrical stimulation were 2.3 ± 0.7 mA in normal animals (mean threshold of 1.8 ± 0.2 mA), 3.5 ± 0.7 mA in arthritic animals (mean threshold of 2.4 ± 0.6 mA) and 3.4 ± 0.8 mA in animals with neuropathy (mean threshold of 2.1 ± 0.5 mA). Mean control number of responses elicited by noxious mechanical stimulation was also very similar in the three groups: 372 ± 47 spikes in normal animals, 298 ± 24 spikes in arthritis and 315 ± 19 spikes in neuropathy. The increment of responses by repetitive electrical stimulation (wind-up index) in the control responses were similar in the three experimental groups: 6.2 ± 1.3 in normal rats, 6 ± 0.9 in monoarthritis and 6.3 ± 1.6 in neuropathy. No significant differences were observed between the threshold intensities or mean control number of responses when the three experimental groups were compared.

**Figure 4:**
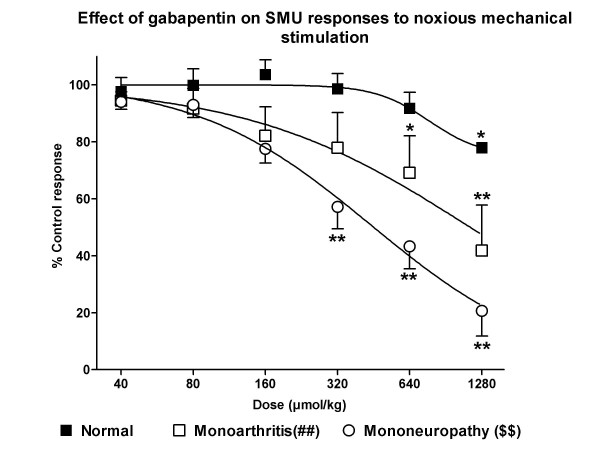
**Antinociceptive effect of gabapentin in responses to noxious mechanical stimulation**. The iv. administration of gabapentin induced a dose-dependent inhibition of responses to noxious mechanical stimulation in arthritic and neuropathic rats, but not in normal rats. The calculated ID50s were of 1,145 ± 14 μmol/kg (200 mg/kg) in arthritis and 414 ± 27 μmol/kg (73 mg/kg) in neuropathy (P < 0.001, two tail unpaired t-test). The effect was still significant 30 min after the administration of gabapentin. Statistical comparison of the regression curves showed a significant difference between normal and arthritic animals (P < 0.01; ##) and between arthritic and neuropathic rats (P < 0.01; $$).*P < 0.05, *P < 0.01, comparison vs. control response with the one-way ANOVA, with the post-hoc Tukey test.

The administration of iv. cumulative doses of gabapentin reduced dose-dependently the SMU responses to noxious mechanical stimulation in arthritic and neuropathic animals, but not in normal animals (Figures 3 and 4). In normal animals, a small reduction of responses (78 ± 2% of control response, P < 0.05) was only observed with the highest dose studied (1,280 μmol/kg). In arthritis, the highest dose studied induced a depression of responses of 41 ± 15% of control (Figure 4; P < 0.01) with a calculated ID50 of 1,145 ± 14 μmol/kg (200 mg/kg). However, the most potent antinociception was observed in the group of neuropathic animals, in which the calculated ID50 was 414 ± 27 μmol/kg (73 mg/kg; P < 0.001 compared to that in arthritic animals), the maximal effect observed was of 20 ± 8% of control response (Figure 4, P < 0.01) and the minimum effective dose was 320 μmol/kg (P < 0.01). Comparison of raw data showed that the effect observed in neuropathic animals was significantly more intense than that in arthritic animals for doses of 320 to 1,280 μmol/kg (P < 0.05 in all cases) and in normal animals for the same doses (320 μmol/kg: P < 0.001; 640 μmol/kg: P < 0.01 and 1,280 μmol/kg: P < 0.05). In addition, the effect observed in animals with monoarthritis was significantly more intense than that seen in normal animals for doses of 320 to 1,280 μmol/kg (P < 0.05 in all cases). Statistical comparison of the regression curves showed a significant difference between normal and arthritic animals (P < 0.01) and between arthritic and neuropathic rats (P < 0.01). As in arthritic rats, the effect of gabapentin was still significant 30 min after administration (data not shown).

**Figure 5:**
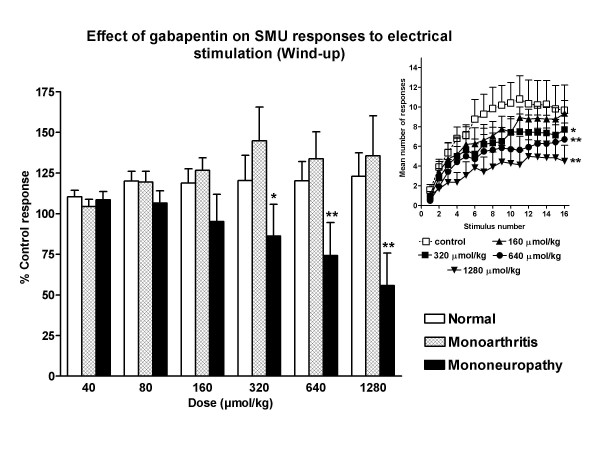
**Effect of gabapentin on wind-up**. The administration of gabapentin dose-dependently reduced the wind-up phenomenon in neuropathic rats with maximal reduction of 55 ± 20% of control (P < 0.01). Gabapentin was not efficacious in the reduction of wind-up in normal and arthritic animals. The figure shows the effect of gabapentin on wind-up as percentage of control (all groups) and as actual number of C-fiber mediated responses (inset, data for neuropathic rats; only some doses are shown for clarity. *P < 0.05, *P < 0.01, comparison vs. control response with the one-way ANOVA, with the post-hoc Tukey test).

All the units studied showed a progressive increment of the number of spikes with repetitive electrical stimulation (wind-up; Figure 1). The phenomenon of wind-up was not significantly reduced by gabapentin in normal and arthritic animals (Figures 3 and 5). However, an important, dose-dependent and significant reduction of wind-up was observed after the administration of gabapentin in animals with neuropathy (Figure 5). In this case, the reduction of wind-up was significant from the dose of 320 μmol/kg (P < 0.05) and the maximal reduction was of 55 ± 20% of control (P < 0.01, Figure 5). The effect of gabapentin on wind-up in neuropathic rats was significantly higher than that in normal animals (P < 0.01) and in arthritic animals (P < 0.01; statistical comparison of the regression curves using the one-way analysis of variance with post-hoc Tukey's multiple comparison test). The depression of wind-up was still significant 30 min after the injection of gabapentin (data not shown).

Finally, the intravenous administration of gabapentin was not followed by any significant change in mean arterial blood pressure. The mean arterial blood pressure before the administration of the drugs was 125 ± 12 mmHg in normal animals, 113 ± 6 mmHg in arthritic animals and 139 ± 7 mmHg in neuropathic animals. After the administration of the highest dose of gabapentin the values were 110 ± 12, 88 ± 4 and 114 ± 3 mmHg respectively.

## Discussion

The main observation made in the present study is the relationship between the antinociceptive effect of gabapentin and the presence and type of spinal cord sensitization. The systemic administration of gabapentin was followed by a slight depression of nociceptive responses evoked by noxious mechanical stimulation in normal non-sensitized animals. The reduction of the responses was significantly more intense in animals with carrageenan-induced sensitization and a virtual full inhibition of responses (less than 25% of control response) was observed in animals with sensitization due to neuropathy. Therefore, our results indicate that in similar experimental conditions, gabapentin is not effective in the reduction of nociceptive responses evoked in normal animals but it is very effective in a situation of spinal cord sensitization. In addition, the antinociceptive effect of gabapentin is more pronounced when hyperalgesia is induced by neuropathy than by articular inflammation. The intensity of behavioral responses in animals with neuropathy, as well as the level of arthritis observed after the administration of carrageenan and, in consequence, the intensity of inflammation-induced sensitization, were similar to those observed in similar experiments performed previously in our lab [34-37]. In addition, thresholds for mechanical and electrical stimulation as well as the control number of responses were similar between groups and similar to those observed in previous studies performed in our laboratory [27,30,36].

It is necessary to consider that the different antinociceptive activity might be due to a different level of anesthesia, a depression of the cardiovascular system or even to a different intensity of stimulation. However, the administration of systemic α-chloralose by perfusion pump ensured a very stable and similar level of anesthesia in the three experimental conditions and so it is not likely that the different intensity of analgesia observed in our experiments was due to a different intensity of anesthesia. In addition, the intravenous administration of gabapentin did not modify significantly blood pressure and, on the other hand, the intensity of stimulation, as well as the number of spikes recorded in the control cycles of stimulation, were very similar in all the experiments (see below and [29,38] for further discussion on these subjects). It seems more likely that the different antinociceptive activity observed by the systemic administration of gabapentin was influenced by the different state of sensitization.

The antinociceptive effects observed after the administration of gabapentin, on the other hand, might have been influenced by the experimental technique. The experiments were performed using the recording of nociceptive withdrawal reflexes as single motor units. This technique has been used frequently by us and other groups in similar experiments, since it allows the recording of direct spinal cord neuronal responses with a minimum of preparatory surgery. The experiments are performed in a less traumatic preparation than that needed in other techniques which, for example, require laminectomy and full exposition of the spinal cord, avoiding artificial and extra nociceptive inputs. The experiments are, in this case, carried out in a much more similar conditions and data are very reproducible [27,29,38-40].

The recording of SMUs allowed us to test for antinociceptive actions of gabapentin, as well as of many other drugs [27,36,37] in different states of sensitization. In addition, the technique allows different protocols of stimulation, combining natural and electrical stimulation. This is important in order to have some evidence as to whether the effect of the drug was located in the periphery, i.e. sensitized nociceptors, or in central areas of the nociceptive system, especially the spinal cord. Noxious mechanical stimulation is transduced by nociceptors, and thus antinociceptive agents acting either in the periphery or within the central nervous system will reduce nociceptive responses. However, drugs acting mainly on nociceptors will have no effect on responses to electrical stimulation. This is because electrical stimulation is not a natural type of stimulus and, therefore, bypasses nociceptors and directly activates afferent axons. In addition, electrical stimulation applied according to the protocol followed in the present conditions, induces the phenomenon of wind-up. In this phenomenon, repetitive electrical stimulation induces a progressive increase of nociceptive responses from spinal cord neurons [24], and is mediated by NMDA [41,42] and NK1 receptors [43]. A reduction of wind-up implies an inhibitory action of the circuitry involved in its generation, which is located in the central nervous system, at spinal cord level [24], although a modulation of the system by higher levels in the CNS is also possible [35]. The important depression of wind-up observed in animals with neuropathy by gabapentin indicates a central action of the drug rather than a peripheral effect. This is supported by previous studies in which an effective action of gabapentin was observed in *in vitro *cortical [4] and dorsal root ganglion neurons [5]. Although the effect was variable and dependent on culture conditions and on the expression of calcium channels [6,22].

However, the fact that the depression of responses to noxious mechanical stimulation in animals with arthritis was not associated to a depression of wind-up indicates that an action of gabapentin on the periphery cannot be rejected. In fact, a peripheral action of gabapentin has been described after intradermal administration of the drug [23], though controversial results are reported in the literature (see [23] and references within for further discussion). Since our experiments, which were made on different states of sensitization but under the same experimental conditions, showed a different action of gabapentin on wind-up, it seems logical to argue that a central effect depends on the type or degree of spinal cord sensitization. This probably means that the effect of gabapentin at peripheral and/or central areas depends on the changes induced in the spinal cord processing of nociceptive system by the sensitization. For example, the expression of calcium channels, as reported by Martin et al 2002 [22] and others [44,45]. Nevertheless, the effect on wind-up is only indicative of a central action and further experiments are needed to reject an action at peripheral sites in the in vivo situation.

## Conclusion

In conclusion, our results show that gabapentin is a potent and effective antinociceptive agent in situations of sensitization caused by arthritis and neuropathy but not in the absence of sensitization. The potency of the antinociception was directly related to the type of sensitization in the present experimental conditions. The effect is mainly located at central sites in neuropathy, however an action on inflammation-induced sensitized nociceptors is also likely.

## Competing interests

The author(s) declare that they have no competing interests.

## Authors' contributions

MMCC carried out all the experiments. JFH conceived the study, participated in its design and coordination and helped to draft the manuscript. All authors have read and approved the final manuscript.

## References

[B1] Tremont-Lukats IW, Megeff C, Backonja MM (2000). Anticonvulsants for neuropathic pain syndromes: mechanisms of action and place in therapy. Drugs.

[B2] Jensen TS (2002). Anticonvulsants in neuropathic pain: rationale and clinical evidence. Eur J Pain.

[B3] Taylor CP, Gee NS, Su TZ, Kocsis JD, Welty DF, Brown JP, Dooley DJ, Boden P, Singh L (1998). A summary of mechanistic hypotheses of gabapentin pharmacology. Epilepsy Res.

[B4] Stefani A, Spadoni F, Giacomini P, Lavaroni F, Bernardi G (2001). The effects of gabapentin on different ligand- and voltage-gated currents in isolated cortical neurons. Epilepsy Res.

[B5] Sutton KG, Martin DJ, Pinnock RD, Lee K, Scott RH (2002). Gabapentin inhibits high-threshold calcium channel currents in cultured rat dorsal root ganglion neurones. Br J Pharmacol.

[B6] Field MJ, Cox PJ, Stott E, Melrose H, Offord J, Su TZ, Bramwell S, Corradini L, England S, Winks J, Kinloch RA, Hendrich J, Dolphin AC, Webb T, Williams D (2006). Identification of the alpha2-delta-1 subunit of voltage-dependent calcium channels as a molecular target for pain mediating the analgesic actions of pregabalin. Proc Natl Acad Sci USA.

[B7] Backonja M, Beydoun A, Edwards KR, Schwartz SL, Fonseca V, Hes M, LaMoreaux L, Garofalo E (1998). Gabapentin for the symptomatic treatment of painful neuropathy in patients with diabetes mellitus: a randomized controlled trial. JAMA.

[B8] Backonja M, Glanzman RL (2003). Gabapentin dosing for neuropathic pain: evidence from randomized, placebo-controlled clinical trials. Clin Ther.

[B9] Rice AS, Maton S (2001). Postherpetic Neuralgia Study Group. Gabapentin in postherpetic neuralgia: a randomised, double blind, placebo controlled study. Pain.

[B10] Serpell MG (2002). Neuropathic pain study group. Gabapentin in neuropathic pain syndromes: a randomised, double-blind, placebo-controlled trial. Pain.

[B11] Patel S, Naeem S, Kesingland A, Froestl W, Capogna M, Urban L, Fox A (2001). The effects of GABA(B) agonists and gabapentin on mechanical hyperalgesia in models of neuropathic and inflammatory pain in the rat. Pain.

[B12] Stanfa LC, Singh L, Williams RG, Dickenson AH (1997). Gabapentin, ineffective in normal rats, markedly reduces C-fibre evoked responses after inflammation. Neuroreport.

[B13] Lu Y, Westlund KN (1999). Gabapentin attenuates nociceptive behaviors in an acute arthritis model in rats. J Pharmacol Exp Ther.

[B14] Field MJ, Oles RJ, Lewis AS, McCleary S, Hughes J, Singh L (1997). Gabapentin (neurontin) and S-(+)-3-isobutylgaba represent a novel class of selective antihyperalgesic agents. Br J Pharmacol.

[B15] Fernihough J, Gentry C, Malcangio M, Fox A, Rediske J, Pellas T, Kidd B, Bevan S, Winter J (2004). Pain related behaviour in two models of osteoarthritis in the rat knee. Pain.

[B16] Nagakura Y, Okada M, Kohara A, Kiso T, Toya T, Iwai A, Wanibuchi F, Yamaguchi T (2003). Allodynia and hyperalgesia in adjuvant-induced arthritic rats: time course of progression and efficacy of analgesics. J Pharmacol Exp Ther.

[B17] Gustorff B, Hoechtl K, Sycha T, Felouzis E, Lehr S, Kress HG (2004). The effects of remifentanil and gabapentin on hyperalgesia in a new extended inflammatory skin pain model in healthy volunteers. Anesth Analg.

[B18] Matson DJ, Broom DC, Carson SR, Baldassari J, Kehne J, Cortright DN (2007). Inflammation-induced reduction of spontaneous activity by adjuvant: A novel model to study the effect of analgesics in rats. J Pharmacol Exp Ther.

[B19] Villetti G, Bergamaschi M, Bassani F, Bolzoni PT, Maiorino M, Pietra C, Rondelli I, Chamiot-Clerc P, Simonato M, Barbieri M (2003). Antinociceptive activity of the N-methyl-D-aspartate receptor antagonist N-(2-Indanyl)-glycinamide hydrochloride (CHF3381) in experimental models of inflammatory and neuropathic pain. J Pharmacol Exp Ther.

[B20] Hanesch U, Pawlak M, McDougall JJ (2003). Gabapentin reduces the mechanosensitivity of fine afferent nerve fibres in normal and inflamed rat knee joints. Pain.

[B21] Carlton SM, Zhou S (1998). Attenuation of formalin-induced nociceptive behaviors following local peripheral injection of gabapentin. Pain.

[B22] Martin DJ, McClelland D, Herd MB, Sutton KG, Hall MD, Lee K, Pinnock RD, Scott RH (2002). Gabapentin-mediated inhibition of voltage-activated Ca2+ channel currents in cultured sensory neurones is dependent on culture conditions and channel subunit expression. Neuropharmacology.

[B23] Todorovic SM, Rastogi AJ, Jevtovic-Todorovic V (2003). Potent analgesic effects of anticonvulsants on peripheral thermal nociception in rats. Br J Pharmacol.

[B24] Herrero JF, Laird JMA, Lopez-Garcia JA (2000). Wind-up of spinal cord neurons and pain sensation: much ado about something?. Prog Neurobiol.

[B25] Seltzer Z, Dubner R, Shir Y (1990). A novel behavioral model of neuropathic pain disorders produced in rats by partial sciatic nerve injury. Pain.

[B26] Gilchrist DH, Allard BL, Simone DA (1996). Enhanced withdrawal responses to heat and mechanical stimuli following intraplantar injection of capsaicin in rats. Pain.

[B27] Mazario J, Gaitan G, Herrero JF (2001). Cicloxygenase-1 versus Cicloxygenase-2 inhibitors in the induction of antinociception in rodent withdrawal reflexes. Neuropharmacology.

[B28] Hargreaves K, Dubner R, Brown F, Flores C, Joris J (1988). A new and sensitive method for measuring thermal nociception in cutaneous hyperalgesia. Pain.

[B29] Herrero JF, Headley PM (1991). The effects of sham and full spinalization on the systemic potency of μ- and k-opioids on spinal nociceptive reflexes in rats. Br J Pharmacol.

[B30] Solano RE, Herrero JF (1997). Cutaneous responsiveness of rat single motor units activated by natural stimulation. J Neurosci Methods.

[B31] Romero-Sandoval EA, Del Soldato P, Herrero JF (2003). The effects of sham and full spinalization on the antinociceptive effects of NCX-701 (nitroparacetamol) in monoarthritic rats. Neuropharmacology.

[B32] Gaitan G, Del Soldato P, Herrero JF (2003). Subeffective doses of dexketoprofen trometamol or nitroparacetamol enhance the effectiveness of fentanyl in responses to noxious mechanical stimulation and wind-up. Eur J Pharmacol.

[B33] Ramos-Zepeda G, Schroder W, Rosenow S, Herrero JF (2004). Spinal vs. supraspinal antinociceptive activity of the adenosine A(1) receptor agonist cyclopentyl-adenosine in rats with inflammation. Eur J Pharmacol.

[B34] Herrero JF, Cervero F (1996). Changes in nociceptive reflex facilitation during carrageenan-induced arthritis. Brain Res.

[B35] Herrero JF, Cervero F (1996). Supraspinal influences on the facilitation of rat nociceptive reflexes induced by carrageenan monoarthritis. Neurosci Lett.

[B36] Curros-Criado MM, Herrero JF (2005). The antinociceptive effects of the systemic adenosine A1 receptor agonist CPA in the absence and in the presence of spinal cord sensitization. Pharmacol Biochem Behav.

[B37] Molina C, Herrero JF (2006). The influence of the time course of inflammation and spinalization on the antinociceptive activity of the alpha2-adrenoceptor agonist medetomidine. Eur J Pharmacol.

[B38] Herrero JF, Solano RE (1999). The antinociceptive effect of the μ-opioid fentanyl is reduced in presence of the α2-adrenergic antagonist idazoxan in inflammation. Brain Res.

[B39] Mazario J, Roza C, Herrero JF (1999). The NSAID dexketoprofen trometamol is as potent as μ-opioids in the depression of wind-up and spinal cord nociceptive reflexes in normal rats. Brain Res.

[B40] Romero-Sandoval EA, Mazario J, Howat D, Herrero JF (2002). NCX-701 (nitroparacetamol) is an effective antinociceptive agent in rat withdrawal reflexes and wind-up. Br J Pharmacol.

[B41] Davies SN, Lodge D (1987). Evidence for the involvement of N-methylaspartate receptors in 'wind-up' of class 2 neurones in the dorsal horn of the rat. Brain Res.

[B42] Dickenson AH, Sullivan AF (1987). Evidence for a role of the NMDA receptor in the frequency dependent potentiation of deep rat dorsal horn nociceptive neurones following C fibre stimulation. Neuropharmacology.

[B43] De Felipe C, Herrero JF, O'Brieny JA, Palmery JA, Doyley CA, Smith AJH, Laird JMA, Belmonte C, Cervero F, Hunt SP (1998). Altered nociception, analgesia and aggression in the mice lacking the substance P receptor. Nature.

[B44] Luo ZD, Chaplan SR, Higuera ES, Sorkin LS, Stauderman KA, Williams ME, Yaksh TL (2001). Upregulation of dorsal root ganglion (alpha)2(delta) calcium channel subunit and its correlation with allodynia in spinal nerve-injured rats. J Neurosci.

[B45] Fehrenbacher JC, Taylor CP, Vasko MR (2003). Pregabalin and gabapentin reduce release of substance P and CGRP from rat spinal tissues only after inflammation or activation of protein kinase C. Pain.

